# O10.4. INCREASED RISKS FOR NON-AFFECTIVE PSYCHOTIC DISORDER AND BIPOLAR DISORDER IN AUTISM SPECTRUM DISORDER

**DOI:** 10.1093/schbul/sby015.256

**Published:** 2018-04-01

**Authors:** Rik Schalbroeck, Fabian Termorshuizen, Jean-Paul Selten

**Affiliations:** 1 Maastricht University; 2 Rivierduinen Mental Health Care Institute; 3 Maastricht University, Rivierduinen Mental Health Care Institute

## Abstract

**Background:**

Young adults with autism spectrum disorder (ASD) appear to be at increased risk for non-affective psychotic disorder (NAPD) and bipolar disorder (BD). However, previous studies have mostly examined the co-occurrence of ASD with NAPD and BD, which is problematic given substantial overlap in symptoms between these disorders. As such, previous risk estimates may have been influenced by diagnostic bias (i.e. NAPD/BD symptoms being mistakenly diagnosed as ASD) or selection bias (i.e. individuals being recognized and/or registered with ASD due to the development of NAPD/BD). In the present study, we used longitudinal data from two Dutch psychiatric case registers to obtain more reliable risk estimates for NAPD and BD among young adults with ASD.

**Methods:**

ASD cases were followed between ages 16 and 35 (n = 17,234). Kaplan-Meier estimates were used to calculate risks for NAPD and BD. We conducted separate analyses to reduce possible bias, taking into account the age of ASD diagnosis (ASD diagnosed before or after age 16) and sequence of diagnoses (ASD before or after NAPD/BD). We conducted prognostic analyses using Cox regression to examine possible risk factors for NAPD and BD in ASD.

**Results:**

ASD cases were at an increased risk for NAPD and BD compared to previously-reported risks in the general population, even when ASD had already been diagnosed at an early age, before a diagnosis of NAPD or BD. Among cases who were diagnosed with ASD at least one year before a diagnosis of NAPD or BD, an estimated 7.90% (95% CI, 6.70–9.31) developed NAPD, whereas 1.35% (95% CI, 0.89–2.04) developed BD, prior to age 36. Prognostic analyses showed that men with ASD were at a relatively greater risk for NAPD, whereas women with ASD were at a greater risk for BD.

**Discussion:**

Young adults with ASD are at an increased risk to develop NAPD and BD, which is not only the result of diagnostic or selection bias. More research is necessary to examine possible mechanisms underlying these risks.

